# The Recent Meeting of the American Dental Association

**Published:** 1876-09

**Authors:** 


### EDITORIAL, ETC.

*The Recent Meeting of the American Dental Association* proved a very interesting and pleasant one, especially as regarded the merits of the papers read, and the harmony and good feeling manifested by the delegates towards one another. The attendance was large, embracing members of the profession in successful practice in foreign countries, as well as very many of the most prominent practitioners of our own.

The first paper read was by Dr. A. H. Brockway, on the "Health of the Dentist," a special committee having been appointed at the previous meeting to prepare a report on this subject. Dr. Brockway contended in his report, that the use of amalgam, a combination of gold and tin, and soft or non-cohesive gold foil, and plastic material when properly employed, instead of adhesive gold foil, acted as important conservators of health and energy, in cases where the strength and physical ability of the practitioner had been greatly taxed.

He also referred to the advantages to the health in the saving of labor by the use of the improved appliances now in use, such as the mallet, engine, &c., in the performance of what in the past were long and laborious operations. Another member of the same committee, Dr. John Allen, of New York City, read a second paper on this subject, in which, after refering to the arduous labors of a large dental practice, and the effects upon the nervous system of the operator as being more depressing than in any other profession, and which he ascribed to the character of certain operations, and the close confinement to which dentists are subject, he dwelt particularly upon the disregard for certain well established, universal and irrevocable laws of nature, among the most prominent being the baneful influence of stimulants and narcotics which are resorted to by some as a relief from nervous prostration. The proper ventilation of op erating rooms was also referred to, and especially recommended as one of the most important adjuncts to physical and mental vigor.

Profs. McQuillen, Shepard, and others also presented interesting and instructive reports. The morning of the last day's session was so occupied by business, that the time assigned by the Association for the reports on Dental Educaation, by Dr. W. H. Morgan, and on Dental Literature by Drs. Allport and W. H. Dwinelle, was not sufficient for their proper presentation and discussion; hence these important subjects received but little attention. Had these subjects been presented earlier in the sessions, to the exclusion of a portion at least of the discussions on some other matters, they no doubt would have received the attention they should always command.

The paper presented by Dr. Dwinelle was an interesting history of the early efforts to establish dentistry as a profession; and that of Dr. Allport a voluminous report on the character of the different dental publications.

Dr. Allport especially deprecated the habit of publishing the same article in more than one journal.

He should, however, have placed the fault where it often properly belongs, namely, with the authors themselves, as frequently the same article is sent by an author to several of the journals at the same time and for the same number without the knowledge of the editors.

The late session of this Association was presided over by Dr. A. L. Northrop, of New York, who for dignity and the qualities incumbent upon a good presiding officer, cannot be surpassed. The veteran Treasurer, Dr. Goddard, of Louisville, Ky., was at his post as usual, and manifested the same degree of activity and interest in the successful management of the pecuniary affairs of the Association as he has done for so many past years. The Chairman of the Committee of Arrangements, Prof. L. D. Shepard, of Boston, by his ability and energy contributed greatly to the success of the meeting, and the members residing in Philadelphia distinguished themselves by their hospitality and kindness to the delegates.

