# Correction

**Published:** 1876-10

**Authors:** 


### EDITORIAL, ETC.

*Correction.*—We cheerfully insert the following communication in justice to the author, Dr. John Allen, of No. 7 West 33rd Street, New York.

MR. EDITOR.—In order to have the records of the late meeting of the American Dental Association authentic, as they appear in our dental journals, permit me to suggest the correction of an error, which I notice in the September number of your Journal—on page 233, you say, "*the first paper read*, was by Dr. Brockway, on the health of the dentists, a special committee having been appointed at a previous meeting to prepare a report on this subject." And in the next sentence you say, "another member of the *same committee*, Dr. John Allen, of New York City, read a *second paper* on this subject." Now the facts are, the committee referred too consisted of Drs. Allen, Morgan and Magill, and the first paper read upon this subject, was the report of the committee by the chairman, Dr. Allen. After which Dr. Brockway read a voluntary paper upon the same subject.

